# Enhanced Biophotocurrent Generation in Living Photosynthetic Optical Resonator

**DOI:** 10.1002/advs.201903707

**Published:** 2020-04-19

**Authors:** Daniel N. Roxby, Zhiyi Yuan, Sankaran Krishnamoorthy, Pinchieh Wu, Wei‐Chen Tu, Guo‐En Chang, Raymond Lau, Yu‐Cheng Chen

**Affiliations:** ^1^ School of Electrical and Electronics Engineering Nanyang Technological University 50 Nanyang Avenue Singapore 639798 Singapore; ^2^ School of Chemical and Biomedical Engineering Nanyang Technological University 62 Nanyang Drive Singapore 637459 Singapore; ^3^ Department of Photonics National Cheng Kung University Tainan City Taiwan; ^4^ Department of Electrical Engineering National Cheng Kung University Tainan City Taiwan; ^5^ Department of Mechanical Engineering National Chung Cheng University Chiayi Taiwan

**Keywords:** bioelectricity, biophotovoltaics, energy coupling, microalgae, optical microcavities, photosynthesis

## Abstract

Bioenergy from photosynthetic living organisms is a potential solution for energy‐harvesting and bioelectricity‐generation issues. With the emerging interest in biophotovoltaics, extracting electricity from photosynthetic organisms remains challenging because of the low electron‐transition rate and photon collection efficiency due to membrane shielding. In this study, the concept of “photosynthetic resonator” to amplify biological nanoelectricity through the confinement of living microalgae (*Chlorella* sp.) in an optical micro/nanocavity is demonstrated. Strong energy coupling between the Fabry–Perot cavity mode and photosynthetic resonance offers the potential of exploiting optical resonators to amplify photocurrent generation as well as energy harvesting. Biomimetic models and living photosynthesis are explored in which the power is increased by almost 600% and 200%, respectively. Systematic studies of photosystem fluorescence and photocurrent are simultaneously carried out. Finally, an optofluidic‐based photosynthetic device is developed. It is envisaged that the key innovations proposed in this study can provide comprehensive insights in biological‐energy sciences, suggesting a new avenue to amplify electrochemical signals using an optical cavity. Promising applications include photocatalysis, photoelectrochemistry, biofuel devices, and sustainable optoelectronics.

## Graphical abstract

A concept of a “living photosynthetic resonator” for amplifying bioelectricity through confinement of microalgae in an optical microcavity is demonstrated. The strong energy coupling between the cavity mode and photosynthetic resonance reveals the potential of exploiting optical resonators to amplify biological photocurrent generation and energy‐harvesting efficiency. Potential applications include photocatalysis, biophotovoltaics, and sustainable optoelectronics.

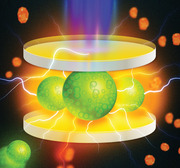

As the world continues to find solutions for the sustainable‐energy production problem, an emerging issue in this field is bioenergy. Bioenergy is renewable energy produced by living organisms, and the photosystems found in plants, cyanobacteria, and algae are considered as potential sources for energy harvesting and bioelectricity generation.^[^ ^1^, ^2^, ^3^ ^]^ Accordingly, the ability to convert natural biological energy to electricity offers a significant potential. Biological photovoltaics (BPVs) are a developing technology that uses the natural process of photosynthesis in organisms to generate electricity.^[^ ^2^, ^4^, ^5^, ^6^, ^7^, ^8^, ^9^, ^10^ ^]^ However, extracting bioelectricity from living photosynthetic organisms such as microalgae remains challenging. The greatest challenge originates from the extremely low transition rate from light to electricity, presumably due to the shielding by membrane systems from chloroplasts.^[^ ^11^, ^12^ ^]^ Recent advances have sought to solve the electrochemical aspects of BPVs, including the design of chambers, nanomaterials, and electrodes^[^ ^11^, ^13^, ^14^, ^15^, ^16^, ^17^, ^18^, ^19^ ^]^ as well as nanophotonic structures to improve the photon‐conversion efficiency.^[^ ^20^, ^21^, ^22^, ^23^, ^24^ ^]^ Instead of enhancing the light‐conversion efficiency,^[^ ^18^, ^21^, ^24^, ^25^, ^26^, ^27^, ^28^, ^29^, ^30^ ^]^ in the present work, we explore the possibility of improving the energy‐transfer efficiency in photosystems through the encapsulation of a living photosynthetic center in an optical microcavity. Whereas microcavities have been utilized to amplify biooptical signals in many ways,^[^ ^31^, ^32^, ^33^, ^34^, ^35^, ^36^, ^37^, ^38^, ^39^ ^]^ they have never been employed to amplify bioelectrical signals.

Bioelectricity generation from microalgae leverage chloroplasts located at the heart of each cell, where the photosynthetic reaction centers are located.^[^ ^9^, ^40^, ^41^, ^42^, ^43^ ^]^ During photosynthesis, two photosystems are involved in microalgae, namely, photosystem II (PSII) and photosystem I (PSI). Each photosystem contains many complex protein pigments that harvest light energy, where the energy is transferred to the special pairs of *chlorophyll a* (Chla) molecule found in the photosynthetic reaction center. The special pair of chlorophylls in PSII is known as P680, while that in PSI is called P700. When excitation energy reaches chlorophylls at the reaction center, electron transfer is initiated through an electron transport chain. Both photosystems exhibit a red fluorescence emission band and dual absorption bands at 420 and 670 nm, which is mainly due to Chla molecules in microalgae (Figure S1, Supporting Information). The huge spectral overlap between fluorescence emission bands (PSII) and absorption band (PSI) is expected to provide high energy transfer efficiency and contribute significantly to bioelectricity generation.

In this study, we propose a novel concept of a photosynthetic resonator to amplify bioelectricity through photosynthesis by confining living microalgae (*Chlorella* sp.) in a Fabry–Perot (FP) microcavity, as shown in **Figure** **1**a. Photosynthetic activity was significantly enhanced by embedding the biophotoelectrochemical cell in an optical resonator. Therefore, the strong energy coupling between the FP cavity mode and photosynthetic resonance (absorption–emission bands in the photosystems) enhances the photocurrent generation (Figure 1b). In this study, we successfully demonstrated the potential of using an optical cavity to enhance bioelectricity generation and bioenergy harvesting for the first time. Biomimetic models and living photosynthesis in algae were explored (Figure 1c) in which the power was markedly increased by more than 600% and 200%, respectively. Exploration of the correlation between fluorescence and photocurrent was conducted to support our significant findings. Finally, we developed an optofluidic device to illustrate the potential application of the photosynthetic resonator. We envisage that the key innovations found in this study offer new possibilities for applications in bioenergy generation, biofuel‐powered devices, and sustainable optoelectronics.^[^ ^10^, ^44^, ^45^ ^]^

**Figure 1:**
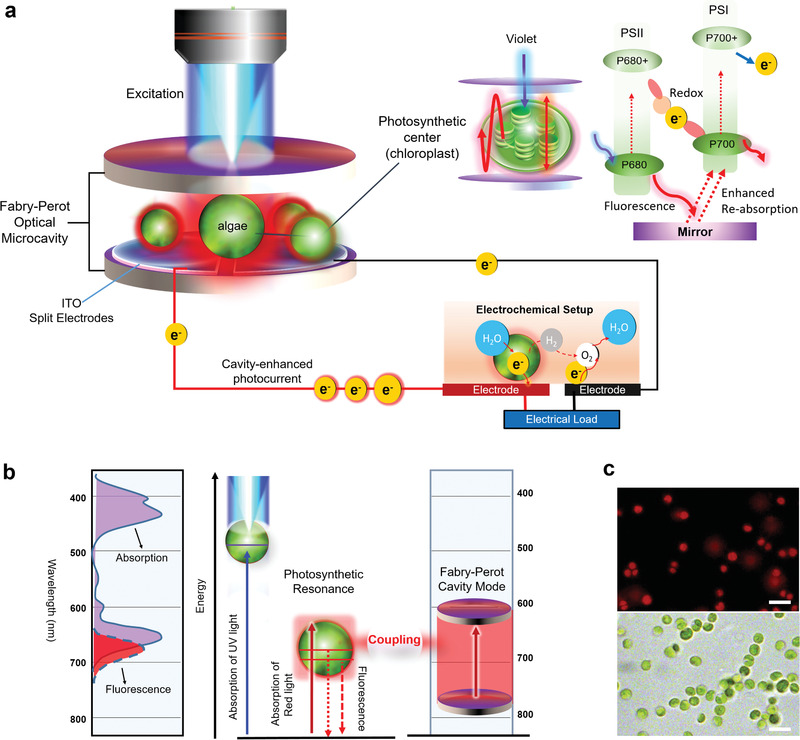
Schematic illustration of the photosynthetic optical cavity. a) The photosynthetic reaction center (biomimetic system or living photosynthesis) is confined between a two‐mirror Fabry–Perot (FP) cavity to enhance photocurrent output. The top‐right panel shows the fundamental mechanism, in which photosystem II and I, located within the chloroplasts undergo violet excitation. Fluorescence emission from PSII (P680) is reflected and optically confined by the FP mirror cavity and reabsorbed by PSI (P700). The bottom‐right panel shows the structure of a biophotovoltaic (BPV) device which comprises of a split ITO electrode to harvest the electrons from the algae. The anode and cathode are connected to a potentiostat via spring loaded clips in a two‐electrode measurement setup. The BPV operates such that the algae take in H_2_O and outputs electrons and H_2_. b) Energy diagram for *chlorophyll a*, representative of photosynthesis in microalgae. The two dashed red lines correspond to the fluorescence generated from two photosystems. Strong coupling between the FP cavity mode and the photosynthetic resonance is designed to enhance the photocurrent efficiency. c) Top image: Fluorescence microscopic image of living *Chlorella* sp. (in vitro) on top of an ITO electrode. The red emission is mainly from *chlorophyll a* inside the chloroplast. Excitation was with violet light (375–450 nm) and fluorescent light collected with a red emission filter. Bottom image: Bright field microscopic image of live *Chlorella* sp. Scale bars, 5 µm.

The concept of photosynthetic microcavity is shown in Figure 1 in which the FP cavity is formed using two highly reflective mirrors (*R* > 99.99%: 600–800 nm) corresponding to the red absorption/emission bands in photosystems. This design enables the red fluorescence emitted from the biological photosystem to be entirely reflected and confined to the reaction centers (P680/P700) to create strong resonance (Figure S2, Supporting Information). To explain the concept of cavity‐amplified bioelectricity, we first investigated the photocurrent generation “without” any optical cavity, as shown in **Figure** **2**. The setup is shown in Figure 2a where a microscope with LED light was used to illuminate a photosynthetic system on an indium tin oxide–polyethylene terephthalate (ITO–PET) electrode. A single piece of ITO–PET film with a split‐electrode design was employed in all measurements. To exploit the novel concept of “photosynthetic microcavity,” living photosynthetic models were studied under different excitation wavelengths. As a proof‐of‐concept, Chla was initially used as a biomimetic system of P700 and P680 to demonstrate the amplified biophotoelectricity in an optical resonator. TiO_2_ nanoparticles were mixed with purified Chla solution to serve as photoanodes, whereas Chla acted as a photosynthesizer in the biomimetic model (Chla–TiO_2_).^[^ ^14^, ^22^, ^46^ ^]^ Figure 2b shows the photocurrent of the Chla–TiO_2_ solutions illuminated under violet, blue, green, and red lights. The photocurrents were measured over three cycles to fully illustrate the photoresponsive nature of Chla and the BPV device. In particular, the strongest peak currents were obtained under the violet (85.0 nA cm^−2^) and red (20.3 nA cm^−2^) lights, which correspond to the two‐absorption peaks of Chla (Figure S1a, Supporting Information). Similar experiments were also performed using *Chlorophyll b* (Chlb) molecules, which is also well‐known for their important role in algae. The results of Chlb–TiO_2_ are shown in Figure 2c where a high photocurrent was obtained only under a violet light because the absorption was relatively low in the other regimes (Figure S1a, Supporting Information). As a control group, photocurrent measurements of pure TiO_2_/water (without chlorophyll) were also performed (Figure S3a, Supporting Information).

**Figure 2:**
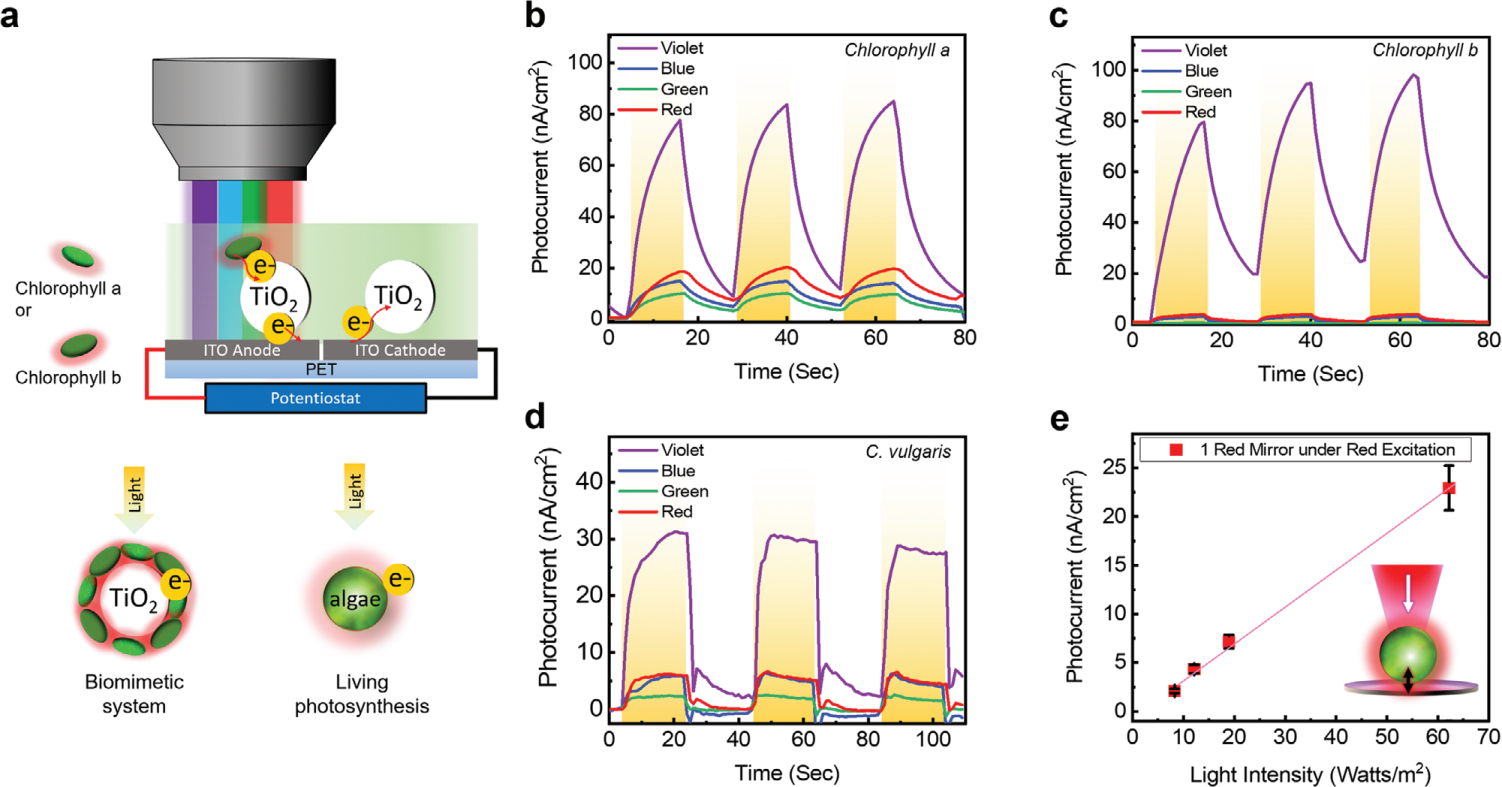
Biomimetic and natural photosynthesis under different excitation wavelengths. a) Schematic diagram showing the setup for the Chla and Chlb biophotovoltaic (biomimetic system of P700 and P680). The solution is a conjugation of Chla or Chlb with TiO_2_ nanoparticles in H_2_O. b) The photocurrent measured when the Chla mixture was deposited into the BPV device and was illuminated by violet, blue, green, and red light. The light had an on–off interval of 15 s. c) The photocurrent measured when the Chlb mixture was deposited into the BPV device under the same conditions as (b). d) Photocurrent over time from *Chlorella* sp. was deposited into the BPV device as per Figure 1a and illuminated by violet, blue, green, and red light for an on–off period of 15 s. e) The algae BPV photocurrent's dependence on light intensity by “red” excitation light with a highly reflective mirror placed beneath (*R* > 99.99%: 600–800 nm). With increasing light intensity, red fluorescence emission is reabsorbed by chloroplasts to generate more photocurrent. Violet excitation: 375–450 nm. Average light intensity: 32.0 W m^−2^.

In addition to the biomimetic system, we next attempted to collect the photocurrent generated from living microalgae (*Chlorella* sp.) using natural photosynthesis. As a control group, photocurrent measurements of a cell culture medium (without algae) were first performed (Figure S3b, Supporting Information). Figure 2d shows the photocurrent generated by *Chlorella* sp. when illuminated under violet, blue, green, and red lights. The peak photocurrent was obtained under violet excitation, followed by the red excitation, which corresponded to the absorption spectrum of the microalgae (Figure S1a, Supporting Information). Overall, the currents were much lower than those of the purified chlorophyll because of the shielding by the complex lipid‐membrane systems. We noted that a substantial amount of photocurrent was generated when excited under red light, indicating the potential of a second absorption band in the red regime. Thus, we investigated the photocurrent when illuminated under different power intensities of “red” light to verify our design, as shown in Figure 2e, by comparing them under the conditions with and without a highly reflective mirror placed underneath the chlorophyll. To our surprise, the photocurrent dramatically increased with the addition of a highly reflective mirror, which laid the foundation for further experiments. This indicates that the increment of red excitation can contribute to more photocurrent generation. We further characterized the photocurrent with different aliquots of *Chlorella* sp. cultures (Figure S4, Supporting Information), in which the increase in volume from 100 to 450 *μ*L, resulted in photocurrents between 23.6 and 35.6 nA cm^−2^ under violet excitation (375–450 nm). The time interval for the light/dark cycle was another factor (Figure S5, Supporting Information).

Subsequent to the generation of photocurrent from the BPV device, we explored the possibility of amplifying the photocurrents within a microcavity. **Figure** **3**a shows the configuration using highly reflective mirrors to confine the fluorescence emission generated from the photosynthetic reaction centers, whereas Figure 3b shows a photograph of the setup. Here, we systematically investigated the photocurrent, power performance, and *I*–*V* curves using one and two mirrors (FP cavity). The ITO thin film adhered to the mirror and was connected to an electrochemical station where the photosynthetic species (Chla or *Chlorella* sp.) was illuminated using violet excitation over three cycles. The absorption and fluorescence spectra of Chla were obtained before and after three cycles of photoelectrochemical measurements (Figure S6, Supporting Information) to ensure quality. The absorbance remained the same, whereas the fluorescence intensity slightly decreased. Figure 3c shows the photocurrent generated from the Chla solution by comparing the results without a mirror, with one‐mirror, and with two‐mirror microcavity setups. Under fixed conditions, a photocurrent of 64.7 nA cm^−2^ was obtained from the Chla solution, whereas the photocurrent increased to 140.2 nA cm^−2^ with the addition of one mirror placed underneath. This surprising result implied that the red‐fluorescence emission can be reflected and reabsorbed by the Chla molecules (Figure S1, Supporting Information). The photocurrent further increased to 174.1 nA cm^−2^ when Chla was sandwiched between two mirrors to create FP resonance. Here, a significant improvement of more than 250% was achieved in the photocurrent when the Chla fluorescence was coupled with the FP‐mode resonance. This ability to substantially enhance the photocurrent by employing FP cavities is further shown in Figure 3d where the *I*–*V* curves are shown. A peak current of 1157.4 nA cm^−2^ was measured from the FP microcavity, and the highly increasing downward gradient in all curves, especially at high currents, indicated the mass transfer limitations and were expected in this design. The peak power was significantly enhanced by more than 600% using the FP two‐mirror cavity, as shown in Figure 3e. Controlled experiments without Chla were also performed to validate the cavity resonance effect (Figure S7, Supporting Information).

**Figure 3:**
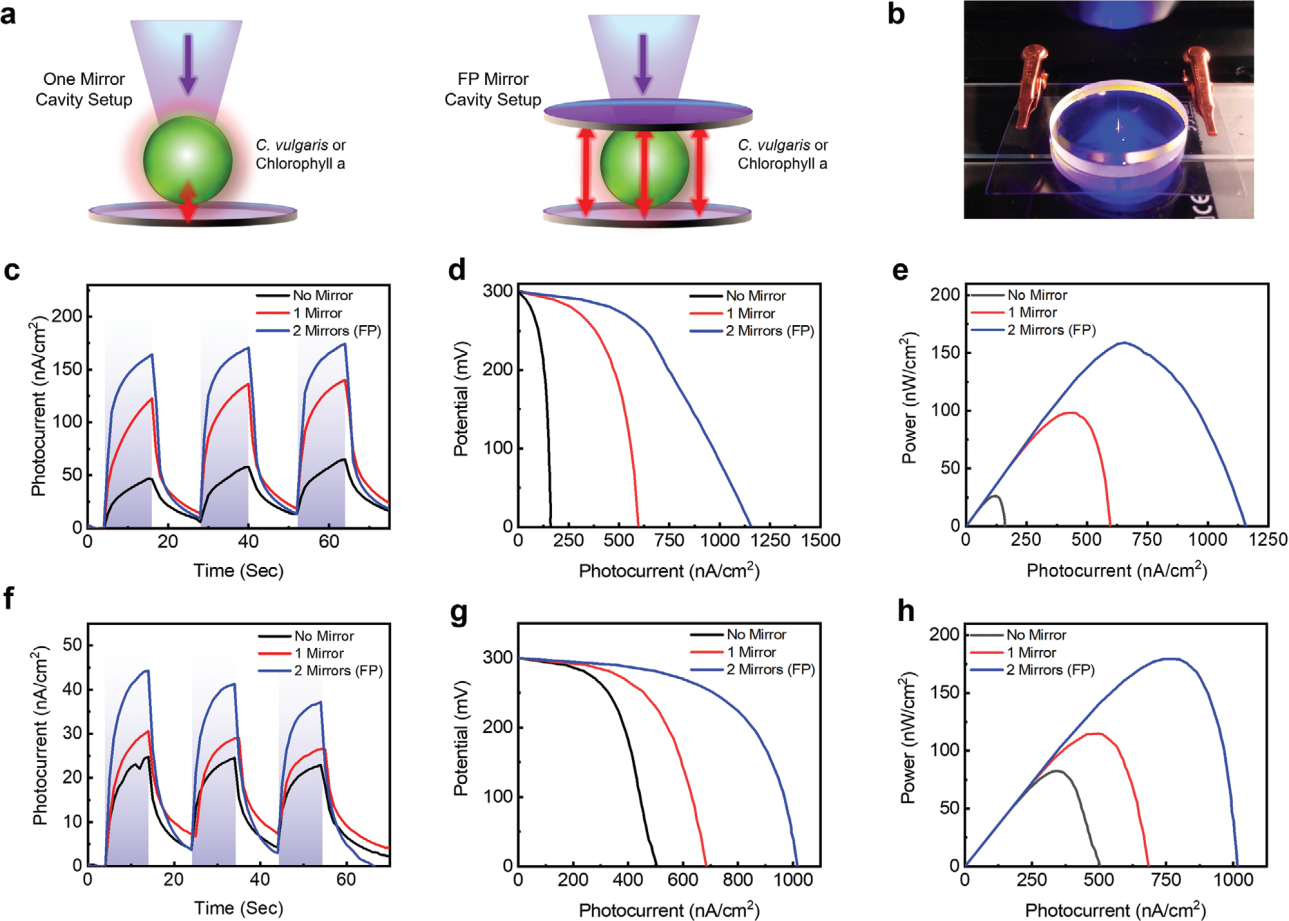
Cavity‐enhanced photocurrent and power generation in photosynthetic cavity. a) Schematic diagram showing the optical cavity setups for the purified chlorophyll (Chla) and *Chlorella* sp. biophotovoltaic cells excited under violet light. b) A photo of the FP‐cavity biophotovoltaic device with *Chlorella* sp. cells being excited by violet light. c) Photocurrent measured versus time from purified Chla molecules illuminated by violet light, without cavity, compared to one and two mirror FP microcavities. d) Applied potential versus photocurrent when Chla was deposited into the BPV device without cavity, compared to one and two mirror FP microcavities when voltages of 0–300 mV were applied. e) Power versus photocurrent for Chla extracted from (d). f) Photocurrent over time from living microalgae (*Chlorella* sp.) illuminated by violet light, without cavity, compared to one mirror and two mirror FP microcavity. g) *I*–*V* curve for *Chlorella* sp. illuminated by violet light, without cavity, compared to one and two mirror FP microcavities. h) Power versus photocurrent for when *Chlorella* sp. was deposited into the BPV device extracted from (g). Light intensity: 32.0 W m^−2^.

Following our discovery of the bioelectricity enhancement from the photosynthetic microcavities, our next goal was to apply this concept to living photosynthesis of *Chlorella* sp. The same setup was applied to *Chlorella* sp., and the results are shown in Figure 3f. A photocurrent of 24.8 nA cm^−2^ was obtained without any mirror, whereas the photocurrent reached 44.2 nA cm^−2^ when the microalgae were confined by a two‐mirror cavity. The control‐group experiments that used a cell culture medium (without algae) confirmed that the enhanced photocurrent was due to the cavity effect (Figure S8, Supporting Information). This result provided us with improvements of close to 200%, which supported the concept of microcavity‐enhanced bioenergy harvesting and generation. It is noteworthy that even without the addition of electron mediators, the effect of the cavity‐enhanced photocurrent was obvious. Furthermore, similar improvements were observed in the polarization curves (Figure 3g) where the increasing downward gradient indicated the mass transfer limitations. Figure 3h shows the power curves of the microalgae with a close to 200% photocurrent enhancement, which correlated well with that shown in Figure 3g. The internal resistances deduced from the FP cavity (299.8 kΩ) were also two times lower than those without a cavity.

To elucidate the fundamental mechanism behind the proposed concept, we simultaneously investigated the correlation between the fluorescence and photocurrent from *Chlorella* sp. First, we studied the fluorescence emission from *Chlorella* sp. by exciting it with different wavelengths (violet, blue, green, and red). The red emission peaks at 680–760 nm were measured resulting from PSII and PSI fluorescence within the chloroplasts. **Figure** **4**a shows that the fluorescence intensity was well correlated with the absorption curve in which the violet and red excitations showed high photocurrent (violet: 20.1 nA cm^−2^; red: 6.6 nA cm^−2^). The trend in the photocurrent also matched the two distinct absorption peaks of *Chlorella* sp. around 420 and 660 nm. Thus, given that the absorption at 660 nm was relatively weak compared with that at 420 nm, a noticeable amount of photocurrent was generated under a red LED excitation.

**Figure 4:**
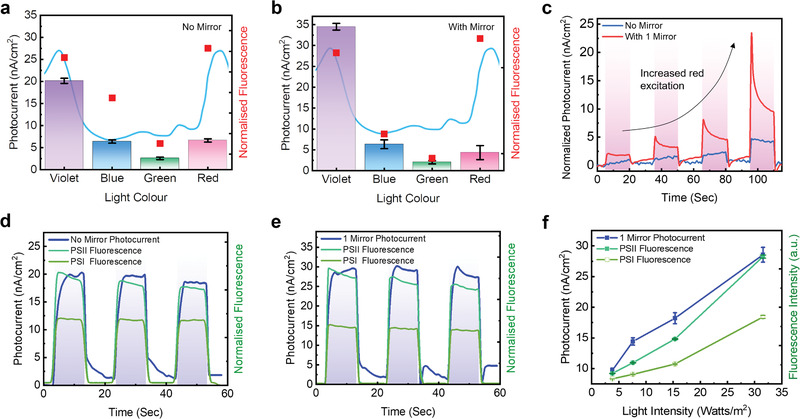
Correlation between photosystem fluorescence and biological photocurrent. a,b) Maximum photocurrent (color bar) and fluorescence (red square) measured from *Chlorella* sp. under violet, blue, green, and red excitation light respectively, a) without any mirror and b) with one highly reflective mirror placed beneath (*R* > 99.99%: 600–800 nm). The light blue curve represents the absorption spectrum for *Chlorella* sp. The trends for both the photocurrent and fluorescence follow the absorbance of algae. c) Photocurrent measured from without mirror and with one mirror from *Chlorella* sp. excited under red light. Red LED light intensity applied: 8.26, 12.12, 18.95, 62.2 W m^−2^.This illustrates that the use of red light does improve the measured photocurrent. d,e) Simultaneous measurement of photocurrent and PSI and PSI fluorescence from *Chlorella* sp. under violet light over time, d) without mirrors and e) with one highly reflective mirror placed beneath. PSII clearly has a larger contribution to the fluorescence and photocurrent. The addition of a mirror affects the fluorescence intensity ratio between PSII/PSI. Light intensity: 32.0 W m^−2^. f) Maximum photocurrent versus PSII and PSI fluorescence measured from *Chlorella* sp. under violet light illuminances with one mirror.

Furthermore, to verify this concept, we placed one mirror beneath *Chlorella* sp. As shown in Figure 4b, the data exhibited a correlation between the absorbance and fluorescence spectra. The violet light showed similar fluorescence intensity with improved photocurrent (≈1.5 times). However, surprisingly, the photocurrent excited by red light was significantly amplified by 2.2 times. Negative controls are also provided (Figure S3c,d, Supporting Information). Figure 4c shows our further verification that the photocurrent generated from *Chlorella* sp. was proportional to the illumination intensity of the red light over time compared with those with and without mirrors. This result suggested that the absorbance at 660 nm played a non‐negligible role in photo‐bioelectricity generation. Therefore, the increment of red excitation (from the fluorescence) also contributed to the increased photocurrent.

Next, we tried to establish on a time basis the relationship between the photocurrent and fluorescence from the PSII and PSI reaction centers. The photocurrent from *Chlorella* sp., along with the fluorescence related to PSII and PSI, is shown in Figure 4d,e. Because the *Chlorella* sp. cells were illuminated with violet light, the fluorescence of both PSI and PSII immediately peaked at each interval. However, the photocurrent response lagged behind the fluorescence. Previously, Anderson et al. found that the photocurrent from *Chlamydomonas reinhardtii* is produced by superoxide anions pumped from the cell membrane.^[^ ^47^ ^]^ This could be the case for *Chlorella* sp., and time would be needed for this event to build up once the photosystems initiate their production in response to light and diffuse to the electrodes. With the addition of one mirror (Figure 4e), the photocurrent only began to decrease after the second interval, suggesting a further lag in the superoxide expression. Finally, we note that the differences in the fluorescence with and without a microcavity over time were interesting. The ratio between the two fluorescence peaks (PSII/PSI) increased with the addition of the mirror. The significantly increased PSII fluorescence intensity may have suggested improved photosynthetic efficiency. The lux level also varied during the measurement of the photocurrent and fluorescence corresponding to PSI and PSII with the mirror. Figure 4f shows the photocurrent and fluorescence (PSI and PSII) under different violet illuminance intensities. PSII demonstrated a much higher intensity than PSI as the photocurrent increased.

Having established that microcavities enhance bioelectrical signals, we expect that the cavity size will affect the coupling efficiency given a fixed algae volume. In this study, we increased the cavity size using bead spacers while maintaining the same number of algae in the cavity. **Figure** **5**a clearly shows that as the cavity length increased by two times, the enhancement of the photocurrent decreased because of the low coupling efficiency between the FP resonance and photosynthesis. Therefore, shrinking the microcavity to a strong coupling regime is expected to improve the bioenergy generation by more than 200%. Without algae, no photocurrent enhancement was observed (Figure 5a, right). Finally, to demonstrate the potential application of the cavity‐enhanced bioenergy concept, we propose a device design with the integration of optofluidics. This process allowed us to obtain a controlled photocurrent by controlling the microalgae flow rate and nutrient flow. Figure 5b shows the optofluidic‐based photosynthetic microcavity. The top layer, which was composed of a SiO_2_ channel, was directly attached to the ITO electrodes. By directly focusing the light into the channel using a 10× objective lens, the maximum current achieved was 197.6 nA cm^−2^. Several cycles were tested, as shown in the right panel in Figure 5b, to demonstrate that living *Chlorella* sp. could withstand the pump power and generate photocurrent.

**Figure 5:**
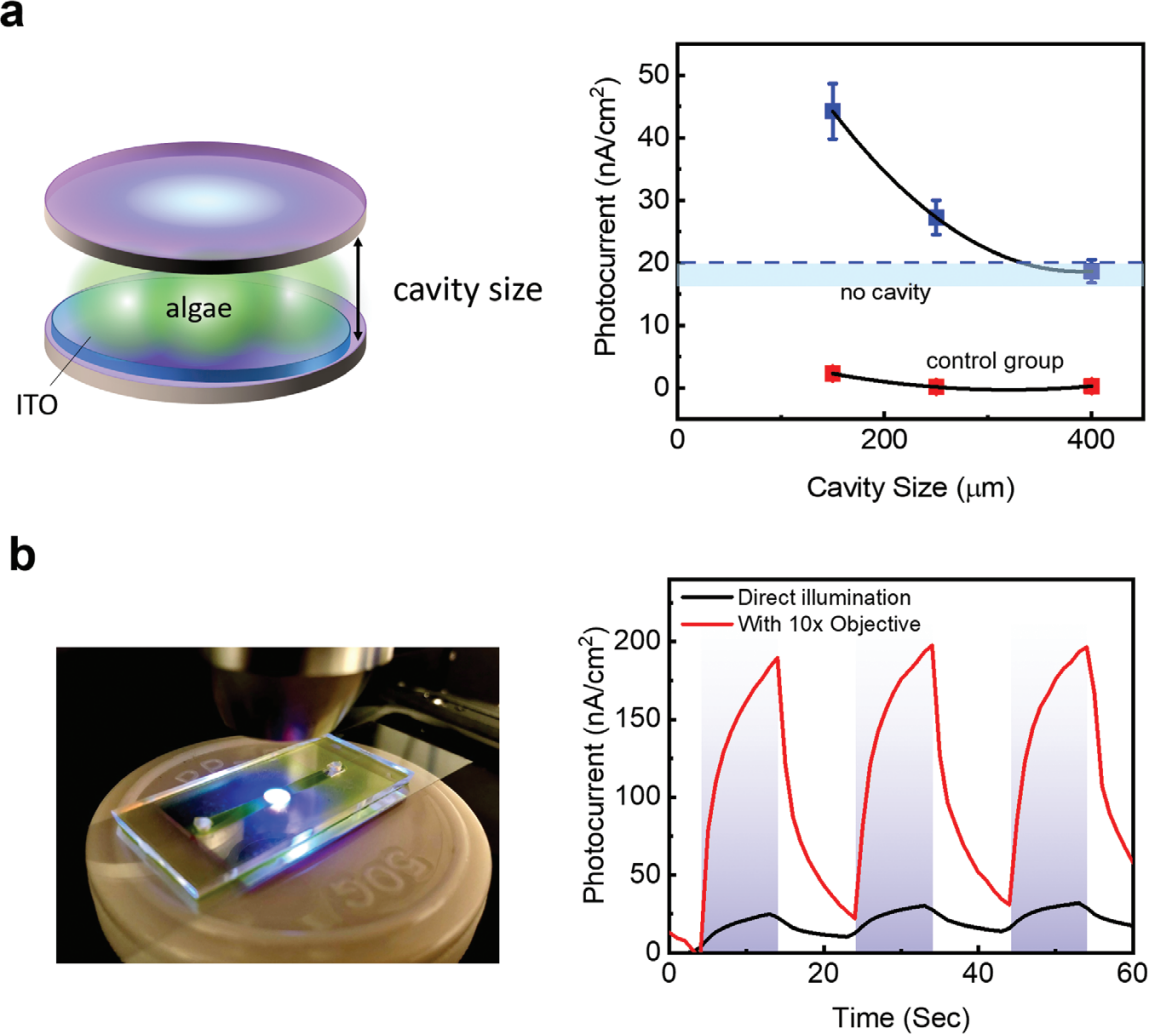
Cavity optimization and optofluidic photosynthetic device. a) Cavity size effect toward photocurrent amplification. Different cavity distances were achieved by inserting silica microbeads with microalgae solution (blue squares). Total cavity distances: 150, 250, and 400 µm. A dashed blue region in the figure shows the average photocurrent without optical cavity. A control group (with only cell medium) was also measured (red squares). Violet light intensity: 32.0 W m^−2^. b) The left photo presents the optofluidic photosynthetic resonator device under violet illumination. The top layer is made of PDMS with inlet/outlet holes whilst underneath is the highly reflective mirror with PET ITO split electrode. A single channel was formed in which living *Chlorella* sp. was injected. The right panel shows the photocurrent over time from *Chlorella* sp. in the microfluidic device illuminated by violet light with no objective and 10× objective.

This study has proposed an entirely novel concept of amplifying biological nanoenergy by confining the photosynthetic centers in an optical microcavity. The energy coupling between the FP cavity modes and photosystem resonance revealed the possibility of exploiting microcavities to amplify the bioelectricity and photocurrent generation in cells or molecules. Both biomimetic systems and living photosynthesis (microalgae, *Chlorella* sp.) were employed as the gain material in which the power was significantly enhanced by more than 600% and 200%, respectively. Systematic studies on the photosystem fluorescence and photocurrent suggested the possible amplification of intracellular energy‐transfer efficiency. Finally, we developed a microfluidic device to illustrate the potential implementation of photosynthetic resonators. In summary, we forecast that the developed bioenergy‐harvesting system will pave the way for new possibilities of bioenergy applications. The discovery in this research also provided a deep insight into the light‐transfer interaction between photochemical synthesis and electricity, which lays the foundation for the development of bioinspired microcavity devices.

Here we identify several future perspectives for the optimization and realization of photosynthetic microcavity devices. First, to optimize the electrochemical materials within the BPV, high‐conductive electrodes need to be applied. For instance, in Figure S9a (Supporting Information), the copper electrodes were made to directly adhere to the mirror cavity, thus lowering the internal resistance of the BPV. A significant improvement of nearly 200% in the photocurrent was achieved owing to the increased conductivity. Second, for optimal energy output, we further propose a parallel design, as shown in Figure S9b (Supporting Information). Instead of using a split electrode similar to that presented in the previous sections (Figures 2 and 3), the electrodes were designed in parallel with the optical cavity resonating direction, and the distance for chemical diffusion between the electrodes should be minimized. Third, by increasing the chip and illumination area, a considerably higher photocurrent of up to microampere levels can be easily obtained. Finally, the developed “photosynthetic microcavity” does not need to be applied only to the species used in this work. This concept can be widely applied to a broad range of living photosynthetic species from the molecular to the microorganism level, such as cyanobacteria, photosynthetic proteins, and biofilms.

## Experimental Section

See the Supporting Information for details of (1) biological material preparation, (2) photoelectrochemical measurements, (3) device design, and (4) optical system setup.

## Conflict of Interest

The authors declare no conflict of interest.

## Supporting information

Supporting Information
