# Supplementary material for: Enhanced Biophotocurrent Generation in Living Photosynthetic Optical Resonator
Source: Adv Sci (Weinh). 2020 Apr 19;7(11):1903707. doi: 10.1002/advs.201903707 (PMC7284217; doi:10.1002/advs.201903707)
Supplement: Supplementary file 1 — Supporting Information [file ADVS-7-1903707-s001.pdf]

Supporting Information

Enhanced Biophotocurrent Generation in Living Photosynthetic Optical Resonator

Daniel N. Roxby [1], *Zhiyi Yuan* [1], *Sankaran Krishnamoorthy* [2], *Pin-Chieh Wu* [3], *Wei-Chen Tu* [4], *Guo-En Chang* [5], *Raymond Lau* [2], and *Yu-Cheng Chen* [1,2] *

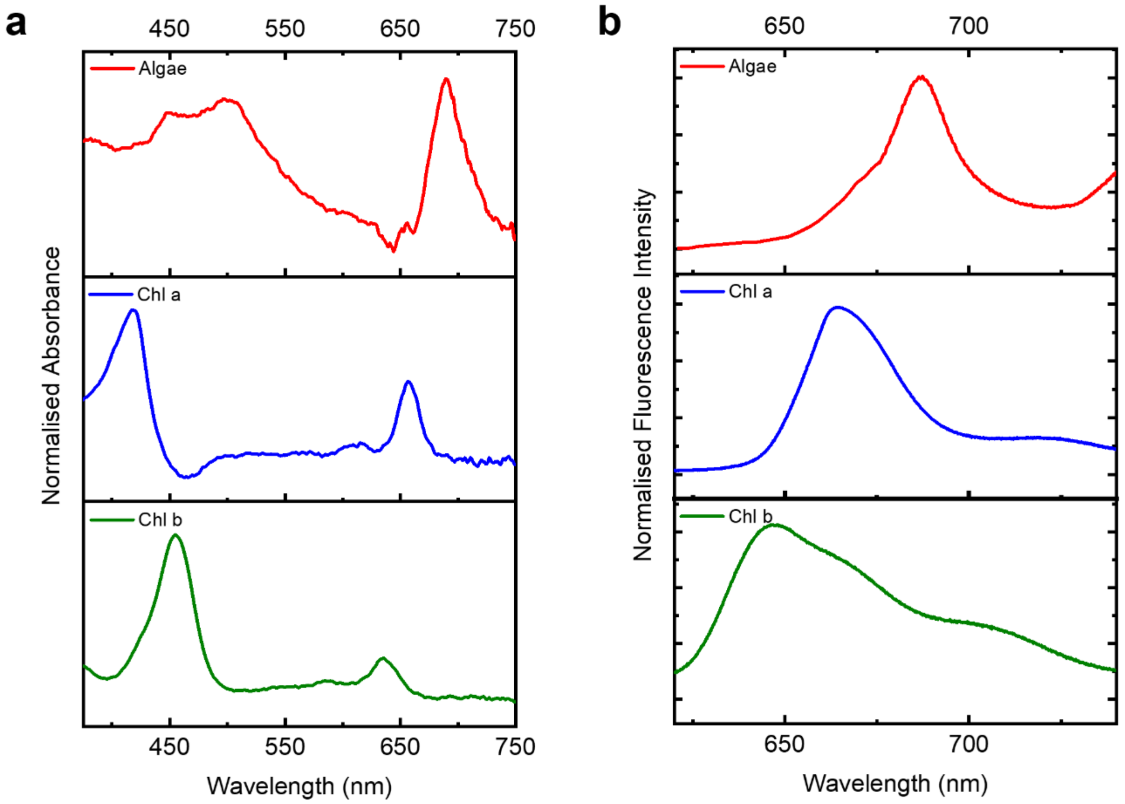

SI Figure S1. a Absorbance for *Chlorella sp.* microalgae in Bold basal medium (top), *Chlorophyll a* in ethanol/water (middle) and *Chlorophyll b* in ethanol/water (bottom). **b,** Fluorescence intensity for *Chlorella sp.* microalgae in Bold basal medium (top), *Chlorophyll a* in ethanol/water (middle) and *Chlorophyll b* in ethanol/water (bottom). *Chlorella sp.* shows broad absorption in the violet wavelengths and a clear absorption peak at 680 nm. Conversely, *Chlorella sp.* has strong fluorescence emission at 680 nm and 730 nm.

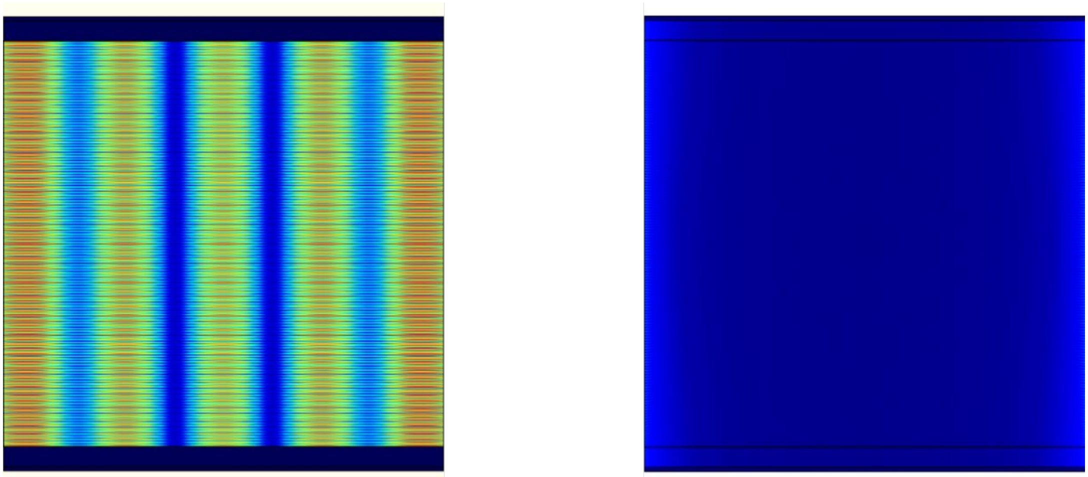

SI Figure S2. Simulation of optical coupling when algae is confined within two high-reflective mirrors (left) and two low-reflective mirrors (right), respectively in the red emission. The top and bottom represent two dielectric mirrors. Strong coupling in the left figure shows the cavity effect when the photosystem fluorescence emission resonates with the dielectric mirror wavelength (600 nm -800 nm).

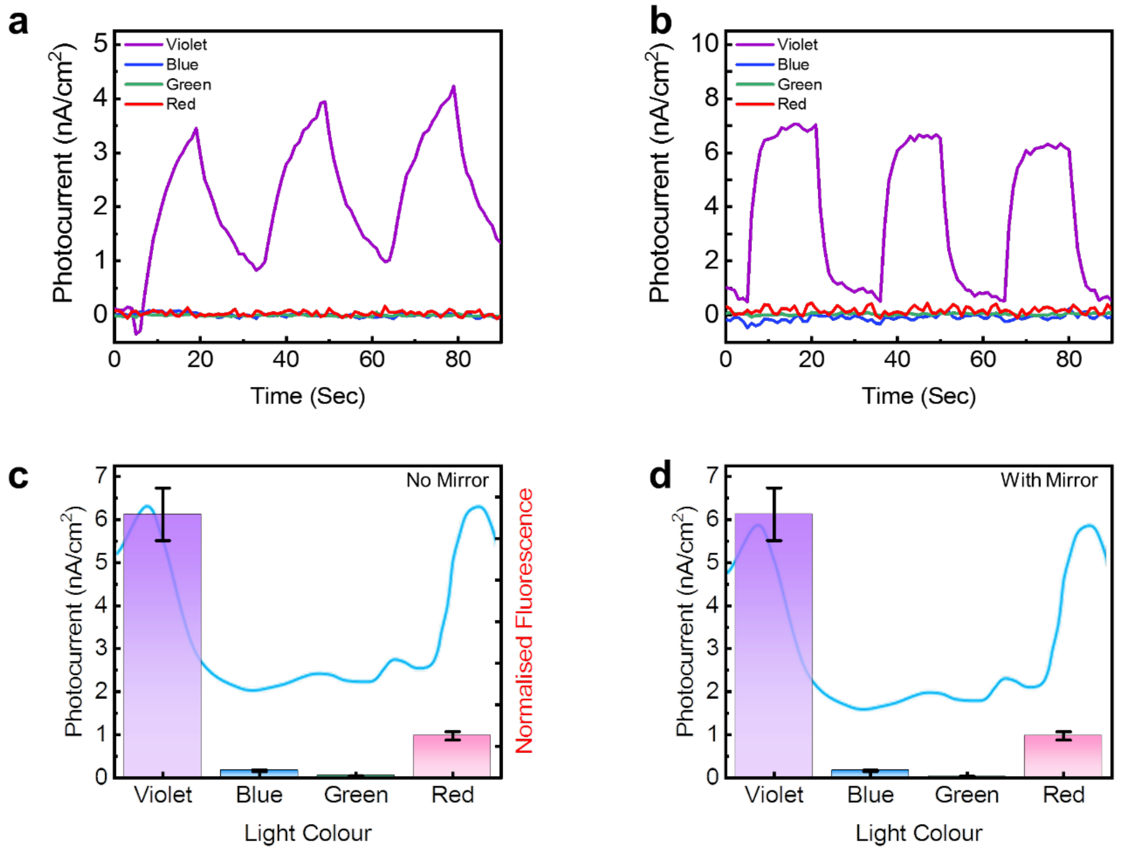

SI Figure S3. a, The photocurrent measured when TiO_2/water (no *Chlorophyll a* or *b*) was deposited into the BPV device and was illuminated by violet, blue, green and red light. The light had an on-off interval of 15 seconds. **b,** Photocurrent over time from when Bold Basal Medium (Chlorella free, only cell medium) was deposited into the BPV device as per Figure 1a and illuminated by violet, blue, green and red light for an on-off period of 15 seconds. **c,** Maximum photocurrent (color bar) measured from Bold Basal Medium (Chlorella free, only cell medium) under violet, blue, green and red excitation light without any mirror. **d,** Maximum photocurrent (color bar) measured from Bold Basal Medium under violet, blue, green and red excitation light with one highly reflective mirror placed beneath (R> 99.99%: 600-800 nm). Violet excitation: 375 nm-450 nm. Violet light intensity: 32 W/m^2. The curves in c and d represents the absorption spectrum.

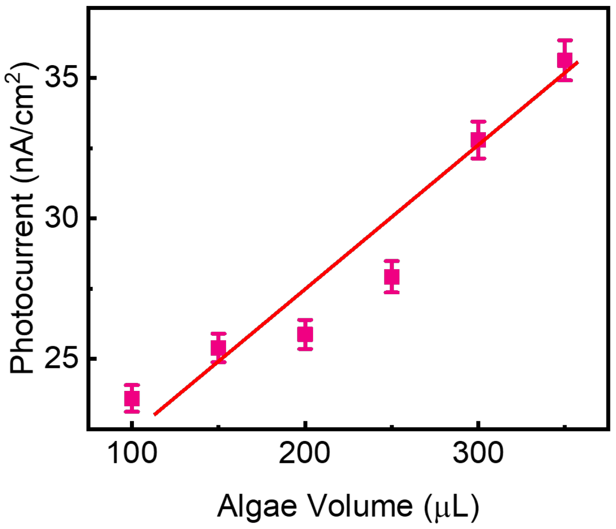

SI Figure S4. The algae BPV photocurrent dependence for different aliquots of *Chlorella sp.* solution is shown for between 100 to 400 μL. With increasing volume, more algae cells are able to be illuminated and generate photocurrent from 23.6 to 35.6 nA/cm^2. Violet light intensity: 32 W/m^2.

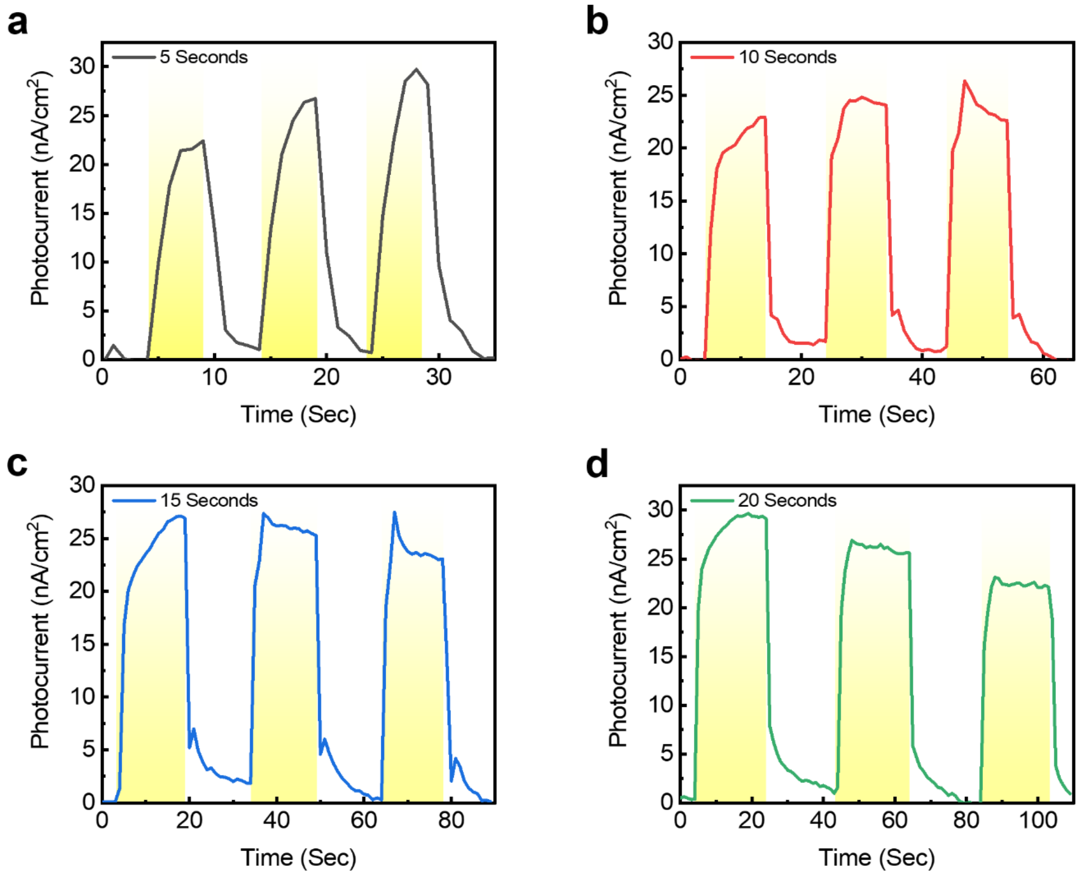

SI Figure S5. a, Violet photocurrent measured from *Chlorella sp.* in a on-off interval of 5 seconds with the split electrode. The peak current for the 3 on intervals increases with each pump of light. **b,** Violet photocurrent with an on-off period of 10 seconds from *Chlorella sp.* with the split electrode setup. The increases between the 3 intervals is less dramatic than that of the 5 second intervals. **c,** Photocurrent measured from *Chlorella sp.* with a 15 second on-off interval in the split electrode setup. With this timing period, the currents between the 3 intervals trend in a decreasing manner across the overall measurement. **d,** Photocurrent measured in a split electrode setup from *Chlorella sp.* with an on-off interval of 20 seconds. Violet excitation: 375 nm-450 nm. Violet light intensity: 32 W/m^2.

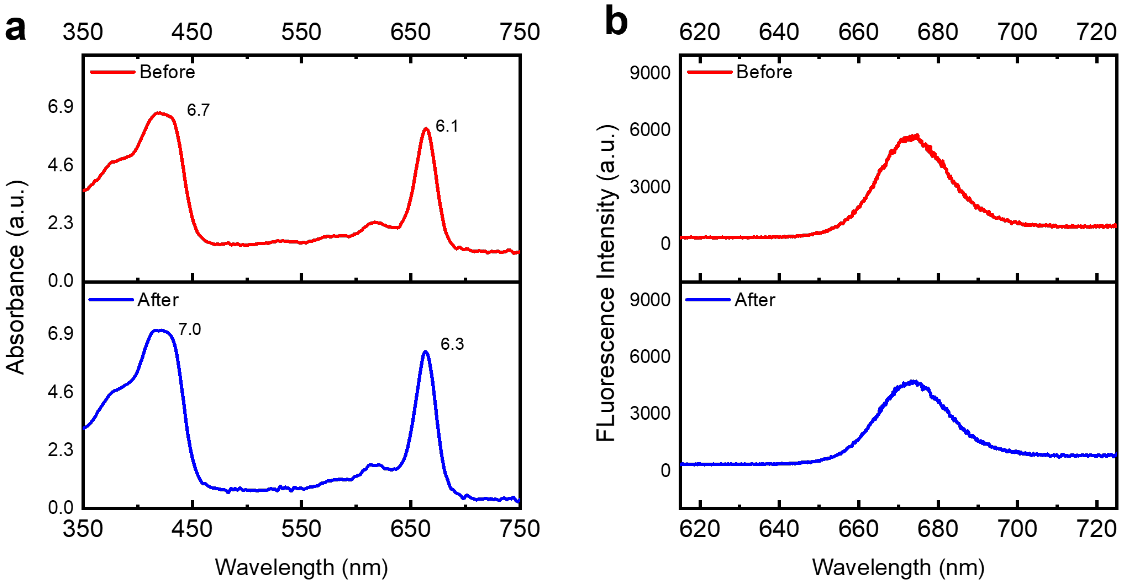

SI Figure S6. a, Absorbance for *Chlorophyll a* in ethanol/water before (top) and after (bottom) undergoing 3 cycles of 15 seconds of violet light illumination. **b,** Fluorescence intensity measured for *Chlorophyll a* in ethanol before (top) and after (bottom) undergoing 3 cycles of 15 seconds of violet light illumination. The ratio between the absorption peak and red absorption peak is 1.09 (before) and 1.1 (after). The fluorescence intensity decayed a little due to possible photobleaching effects after 3 cycles. Violet light intensity: 32 W/m^2.

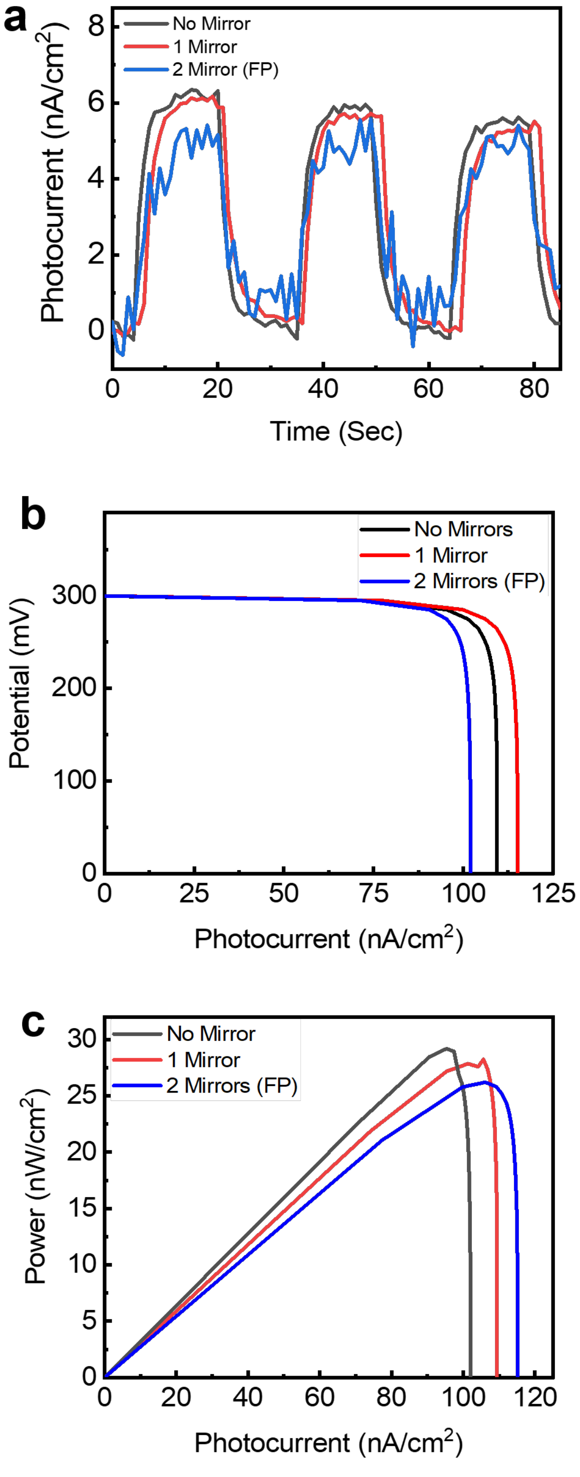

SI Figure S7. a, Photocurrent over time from TiO_2/water (without *Chlorophyll a* or *b*) by violet light, without cavity, compared to one mirror and two mirror FP microcavities. **b,** I-V curve for TiO_2/water illuminated by violet light, without cavity, compared to one and two mirror FP microcavities. **c,** Power vs photocurrent for when TiO_2/water was deposited into the BPV device and extracted from b. Violet light intensity: 32 W/m^2.

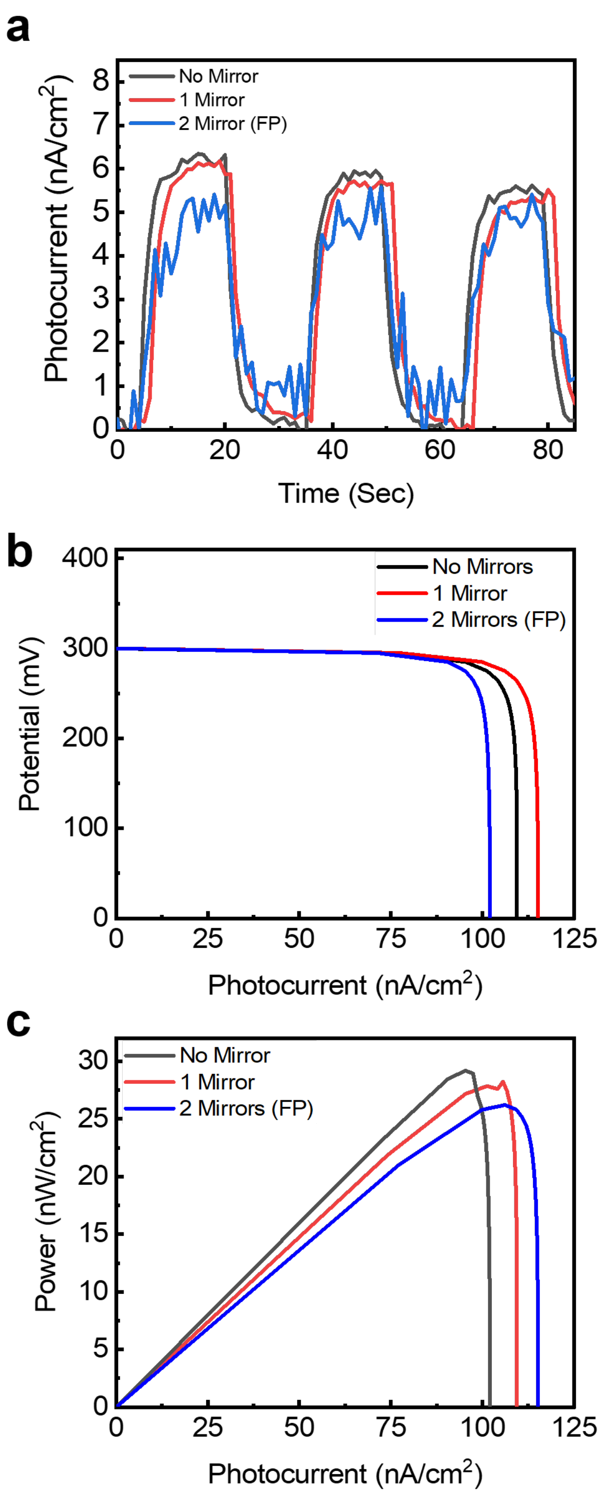

SI Figure S8. a, Photocurrent over time from Bold Basal Medium (without *Chlorella sp.*) illuminated by violet light, without cavity, compared to one mirror and two mirror FP microcavities. **b,** I-V curve for Bold Basal Medium illuminated by violet light, without cavity, compared to one and two mirror FP microcavities. **c,** Power vs photocurrent for when Bold Basal Medium was deposited into the BPV device extracted from b. Violet light intensity: 32 W/m^2.

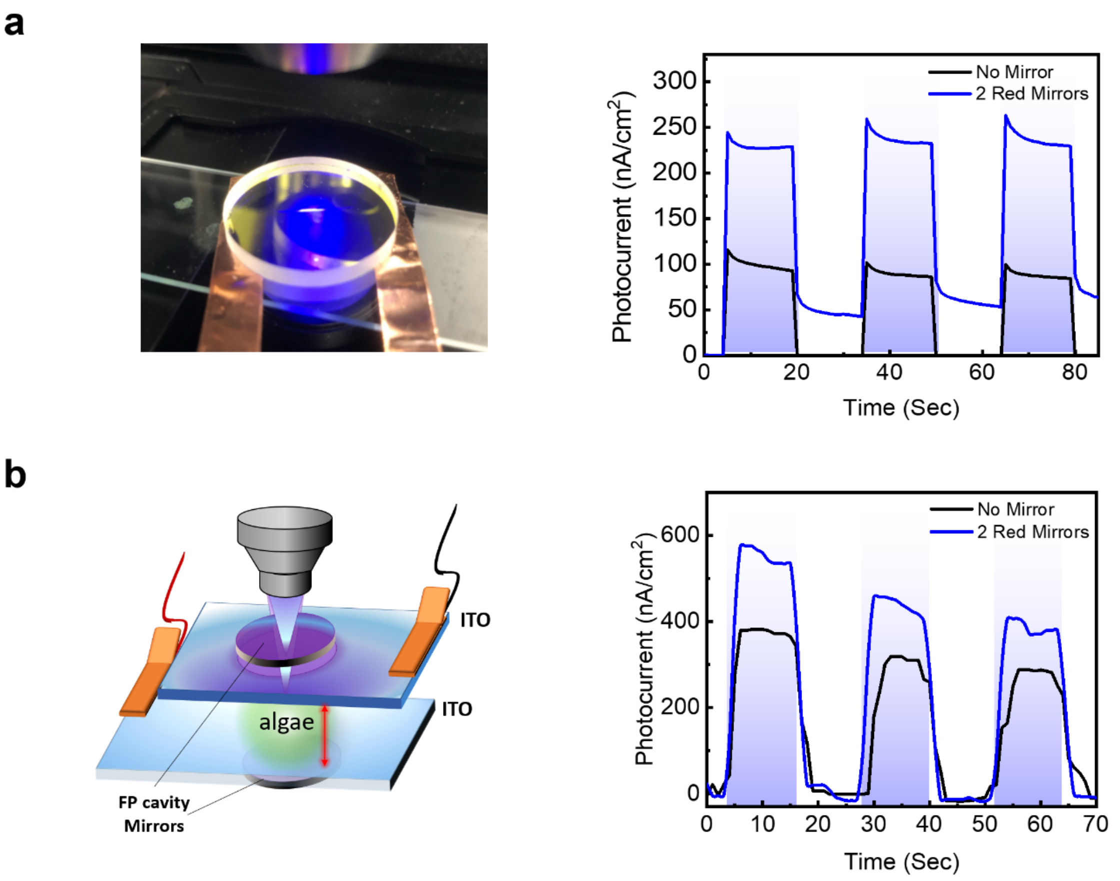

SI Figure S9. a, Photo of the copper tape device in which copper tape is attached to the bottom mirror and acts as the electrodes (left). *Chlorella sp.* is then deposited and the top mirror is added to create the microcavity before violet light is shone to excite the algae. The main advantage of this design is that the copper tape is significantly more conductive than the ITO electrodes. Photocurrent overtime for the from *Chlorella sp.* within the copper tape electrode-based device with no mirror and two mirror FP microcavity, excited by violet light (right). The use of more conductive copper tape, increases the overall current production, however, between the no mirror and FP microcavity, the latter provides up to 3x more photocurrent than the former. **b,** Schematic of the parallel electrode device with *Chlorella sp.* and two mirror microcavity (left). Two parallel sheets of ITO are sandwiched together within a FP microcavity. This minimizes the distance travelled by chemicals between electrodes, lowering the overall fuel cell internal resistance. Photocurrent over time from *Chlorella sp.* within the parallel electrode device with no mirror and FP microcavity, illuminated by violet light (right). As can be seen, the photocurrent is significantly higher in the parallel setup, as compared to the split electrode setup. This current is further enhanced by the FP microcavity. Note that the photocurrent increase with the addition of a FP cavity was less than that in the configuration in Fig. 3a, due to the increased cavity size. However, this can be resolved by depositing a thin ITO layer (~100 nm) on both sides of the FP cavity. More importantly, this result further verifies that microcavity confinement can drastically improve bioelectricity generation. Violet excitation: 375 nm -450 nm. Violet light intensity: 32 W/m^2.

Methods and Materials

Biological Material Preparation

Chlorella sp. (ATCC14854) was grown as per a previously described growth method[16]. Cells were maintained under sterile conditions in an Erlenmeyer flask shaking in an incubator at 150 RPM and a temperature of 25°C. Light for the growth of *Chlorella sp.* was provided by an 8W fluorescent bulb with a measured photon density of 22.73 umol/s/m^2 during a light:dark cycle of 14:10. Prior to each photoelectrochemical measurement, under sterile conditions, 15mL of cells at a density of 5.92×10^7 cells/mL, was spun down at 4000 RPM for 8 minutes and the cells were separated out from the solution. 2 mL of Bold Basal Medium (Thermo Fisher Scientific) was prepared as per instructions with sterile water, then added to the cells, homogenized with a vortex and used as is on that day. Absorbance measurements were also conducted with this same solution.

Chla and Chlb were purchased from Sigma Aldrich in powder form and dissolved in ethanol, then diluted with water to a concentration of 0.5 mM. Next 250 µL of this mixture containing either Chla or Chlb, was then added to 125 µL of H_2O with 125 µL of 100nm sized TiO_2, homogenized with a vortex, then used as is.

Photoelectrochemical Measurements

The photocurrent produced by *Chlorella sp.* was measured within the setup as per Fig. 1a. PET ITO films (Nanocs) with a resistance of 50 Ω/cm were used as the electrode material. To create an anode and cathode, a small cut was made with a knife into the ITO layer, but not into the PET. This was verified with the continuity tester of a multimeter and the surface area of each electrode was approximately 1.872 x 2.5 cm. Toothless crocodile clips with prototyping wire were then used to connect to the potentiostat leads. 50 µL of the *Chlorella sp.* BBM mixture was deposited onto the ITO electrodes, and in order to create a thin film in which the algae were spread across and in close proximity to the electrodes, a glass cover slip was used. Upon preparation of the device, it was inserted into the microscope platform and the light turned on and current being recorded to aim the beam and ensure the device was operating correctly prior to formal measurements. The light beam had a circular area of 1.77 cm^2. Each experiment was repeated at least three times where each time a new deposit of algae onto the ITO was prepared and each deposit was from the same algae culture. All experiments measuring photocurrent over time used a Zahner Zennium potentiostat in chronoamperometry mode, set to 0 V to maximize current without biasing it. Polarization and power curves were collected with the

same potentiostat setup in linear sweep voltammetry mode, at a scan rate of 0.1 mV/s with no dwell time. The internal resistances were deduced by finding the resistance at the peak power, and the power determined by multiplying the voltage and current (Ohm's law). Each experiment was run in open air, at room temperature.

Device Design

The device was composed of two highly reflective mirrors (Edmund Optics, R>99.9% in 600 nm - 800 nm), in which an ITO-PET film or copper tape (120 µm thick) was adhered on the bottom mirror, as previously described. The potentiostat was then connected via clips and wire, in a two-electrode setup, and a microscope with LED was used to illuminate the photosynthetic apparatus. When using chlorophylls, either Chla or Chlb was mixed with H_2O and TiO_2 as previously described to assist in the mediation of electrons between the chlorophylls and electrodes. Photocurrent was measured in the absence of TiO_2, however it was unstable. Whether using *Chlorella sp.* or chlorophylls, once the mixture was deposited on to the ITO electrodes, a glass coverslip was used to disperse the solution evenly along the electrodes, due to the hydrophobic nature of ITO-PET. This also allowed for the photosynthetic apparatus to be close to the electrode, without the need for lengthy gravity-based depositions and for the beam to illuminate more of the solution on the anode. When the Chl mixtures were deposited into the device, they were either illuminated by violet, red, blue or green light. The light was turned on for approximately 15 seconds, then off for 15 seconds, and repeated for a total of three cycles, to fully illustrate the photo-responsive nature of the chlorophylls and BPV device.

Optical System Setup

To illuminate the photosynthetic apparatus, the device was placed atop a microscope slide and mounted on a Nikon NI-E microscope with no objective set, unless otherwise stated. Light was generated by an attached SOLA light engine and filtered for different colors by filter cubes, as appropriate. Both bright field and fluorescent images were collected with a ToupTek CCD camera, while fluorescence spectrums were collected with an Andor Kymera 328i /Newton 970 EMCCD spectrophotometer. The absorbance spectra in SI was collected with Implen N80 nanophotometer.
